# Editorial: Extended Data at *eNeuro*

**DOI:** 10.1523/ENEURO.0103-17.2017

**Published:** 2017-03-29

**Authors:** Christophe Bernard

Dear friends and colleagues,

From launch, we decided that all scientific arguments essential to supporting a story need to be included in the manuscript when published. We started with the option for source data, which allowed for supporting small datasets. In some instances, however, larger extensions of datasets are required to compliment the results, which was a limitation of source data files.

We are pleased to announce that authors will now be able to include larger datasets as extended data to the figures or tables of the paper. We see these data as a natural extension of what constitutes the main message of the results. These data could include a video of a behavior, a control experiment, a table with a list of proteins, etc.

Extended data will be peer reviewed, and during the consultation process, it will be determined whether or not extended data are essential for the story. We have recently upgraded our web platform to accommodate these larger data files. Authors should first consider depositing larger data files in a public repository to help maintain anonymity in compliance with the double-blind review system. If a repository that maintains anonymity to comply with the double-blind review system is not available for storage, then the author can upload extended data (up to 20 MB) with their manuscript.

The extended data policy can be found here. We believe that this feature constitutes a major improvement for the publication of our research, by providing readers more information without disrupting the logical flow of the scientific story.

